# A Randomized, Double-Blind, Contralateral Eye Study Comparing the Clinical Outcomes of Two Types of Silicone Hydrogel Bandage Contact Lenses After Transepithelial Photorefractive Keratectomy

**DOI:** 10.3390/bioengineering13010039

**Published:** 2025-12-29

**Authors:** Ling Wang, Jiajia Jin, Lu Yan, Kaiyan Huang, Shihao Chen

**Affiliations:** 1 National Clinical Research Center for Ocular Diseases, Eye Hospital, Wenzhou Medical University, Wenzhou 325027, China; 13857737840@eye.ac.cn (L.W.);; 2 State Key Laboratory of Ophthalmology, Optometry and Visual Science, Eye Hospital, Wenzhou Medical University, Wenzhou 325027, China; 3 National Engineering Research Center of Ophthalmology and Optometry, Eye Hospital, Wenzhou Medical University, Wenzhou 325027, China; 4 Laboratory of Novel Optoelectronic Technology for Ophthalmic Devices (NOTOD), School of Ophthalmology and Optometry, School of Biomedical Engineering, Wenzhou Medical University, Wenzhou 325027, China

**Keywords:** transepithelial photorefractive keratectomy (TPRK), silicone hydrogel, bandage contact lens, Lotrafilcon A, Balafilcon A

## Abstract

Background: To compare clinical outcomes of Lotrafilcon A and Balafilcon A silicone hydrogel bandage contact lenses (BCLs) following transepithelial photorefractive keratectomy (TPRK). Methods: A randomized, double-blind, contralateral eye study enrolled 41 TPRK patients (82 eyes), with each eye randomly assigned one BCL type. Assessments included uncorrected (UDVA) and corrected (CDVA) distance visual acuity, ocular pain and irritation, epithelial healing, limbal and conjunctival hyperemia, lens mobility, and the amount of protein deposition on the BCLs. Results: Postoperative day 1 pain score was lower in Group A (2.80 ± 2.35) than in Group B (4.44 ± 2.46, *p* = 0.003). Group A had significantly less protein deposition (day 3: 9.92 ± 9.82 vs. 25.75 ± 9.86 μg, *p* < 0.001; day 4: 9.47 ± 10.06 vs. 32.60 ± 16.71 μg, *p* = 0.005). No statistically significant differences were observed between the two groups in terms of corneal epithelial defect area, corneal epithelial healing time, UDVA, CDVA, limbal or conjunctival hyperemia, and lens movement. Conclusions: Lotrafilcon A outperformed Balafilcon A in reducing ocular pain, foreign body sensation, and protein deposition, suggesting that Lotrafilcon A may be a more suitable therapeutic BCL option following TPRK.

## 1. Introduction

Photorefractive keratectomy (PRK) is a proven effective excimer laser technique for the correction of ametropia [1]. Compared with laser in situ keratomileusis (LASIK), PRK preserves a thicker postoperative stromal bed and avoids flap-related complications. However, it carries risks of postoperative pain, haze, delayed corneal epithelial healing, and prolonged visual recovery [2,3,4]. Transepithelial PRK (TPRK) builds upon the principles of PRK [5,6,7,8], removing corneal epithelium and anterior stroma in a single step using excimer laser without mechanical or alcohol-based epithelial debridement. This approach improves intraoperative comfort, reduces corneal trauma, and alleviates postoperative discomfort [9,10], while potentially improving epithelial healing and minimizing haze compared to traditional PRK methods [9,11]. Refractive surgery innovations eliminating corneal flaps avoid iatrogenic aberrations, preserve stromal tissue, maintain biomechanical stability, and reduce long-term corneal risks [12,13]. Despite these advances, TPRK may still cause ocular irritation, pain, and slower visual recovery [10,14], underscoring the need to refine postoperative protocols and patient satisfaction.

Since the late 1960s, bandage contact lenses (BCLs) have been primarily used to protect the cornea after injury. They also demonstrate efficacy in pain relief, corneal epithelial healing promotion, corneal protection, and visual acuity improvement [15], particularly after excimer laser keratectomy [16,17,18,19,20,21,22]. Additionally, BCLs help maintain corneal hydration and prevent postoperative dry eye symptoms [23,24]. Continuous BCL wear for 3–7 days post-PRK has become standard clinical practice [25,26]. Silicone hydrogel BCLs offer superior oxygen permeability compared to hydrogel counterparts, mitigating hypoxia-related complications and fostering optimal corneal epithelial healing and regeneration [27].

Several silicone hydrogel contact lenses are currently in clinical use. Among them, Lotrafilcon A (Air Optix Night & Day, Alcon Laboratories, Fort Worth, TX, USA), Balafilcon A (PureVision, Bausch & Lomb, Bridgewater, NJ, USA), and Senofilcon A have been approved by the U.S. Food and Drug Administration (FDA) for use as BCLs [28]. These lenses differ in terms of their material composition and structural design, resulting in variations in oxygen permeability, water content, elastic modulus, thickness, diameter, edge contour, and resistance to protein deposition [29,30]. These parameters can influence ocular comfort and corneal healing following surface ablation surgery [31,32].

The first-generation silicone hydrogel contact lenses, Lotrafilcon A and Balafilcon A, have been shown to be both safe and effective as BCLs [33,34]. They continue to be widely utilized in the treatment of various ophthalmic diseases, particularly ocular surface disorders [35,36]. In an effort to enhance ocular comfort, the manufacturer has recently introduced an updated version of Lotrafilcon A BCL that incorporates an AQUA moisturizing agent. Given that silicone hydrogel materials vary in chemical composition (e.g., cross-linker density, silicone monomer ratio) and physical properties (e.g., water content, Dk/t value), we hypothesize that these structural variations may influence interactions with ocular tissues—such as modulating inflammatory responses, protein adsorption kinetics, or epithelial cell adhesion. For instance, the higher oxygen permeability (Dk/t) in Lotrafilcon A compared to Balafilcon A may reduce inflammation caused by corneal hypoxia, while differences in surface chemistry could alter protein deposition patterns, thereby impacting pain perception and healing dynamics. This study aims to evaluate the clinical performance of the new-generation Lotrafilcon A (Air Optix) and Balafilcon A (Pure Vision) BCLs.

## 2. Materials and Methods

A randomized, double-blind, contralateral study was conducted on 82 eyes of 41 consecutive patients who underwent TPRK at the Refractive Surgery Center of the Eye Hospital of Wenzhou Medical University from October 2018 to March 2019. Inclusion criteria were as follows: myopia of ≤10.00 diopters (D), astigmatism of ≤2.00 D, anisometropia ≤1.00 D in spherical equivalent refraction (SER), and corrected distance visual acuity (CDVA) of 0 (LogMAR) or better. Patients with ocular disease, amblyopia, or a history of previous eye surgery were excluded from the study. The study protocol was approved by the Ethics Committee of Wenzhou Medical University Eye Hospital (the approval number is Y2017-063) and adhered to the tenets of the Declaration of Helsinki. Prior to participation, all patients’ written informed consent was obtained.

All surgeries were conducted by the same experienced surgeon (S.C.). The procedures were performed using the Amaris 750 excimer laser (Schwind, Kleinostheim, Bavarua, Germany) in TPRK mode, with a therapeutic BCL placed on the treated eye immediately after the procedure. The patients’ two eyes were randomly assigned to two groups based on a randomization table. One eye was fitted with Lotrafilcon A BCL (Group A), while the fellow eye was fitted with Balafilcon A BCL (Group B). Both the patients and the examiner were masked of which lens was applied to each eye. The characteristics of the two types of BCLs are provided in Table 1.

**Figure 1:**
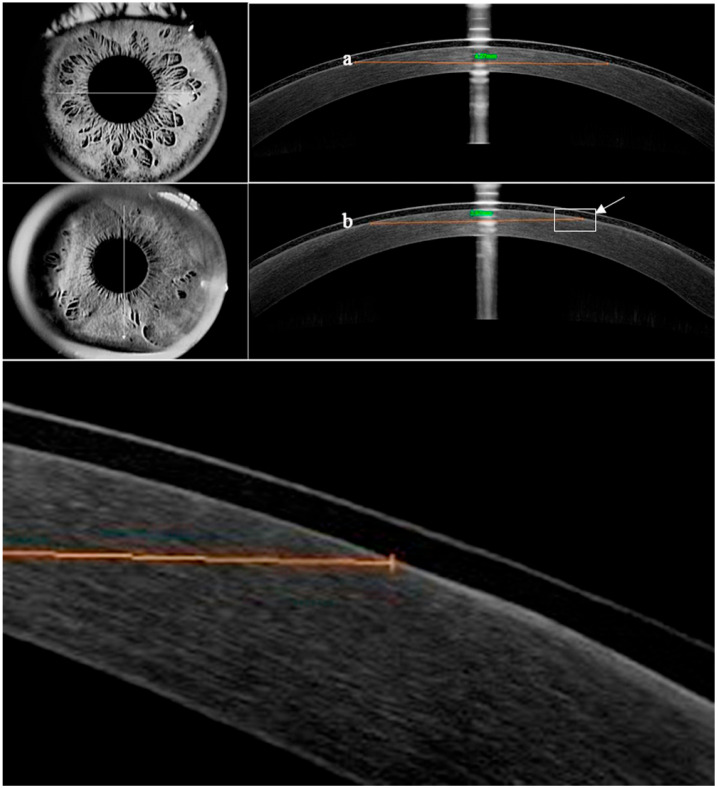
Horizontal (**upper image**) and vertical (**middle image**) linear optical coherence tomography (OCT) scans were taken through the corneal apex, with the patient fixating straight ahead. The (**a**) and (**b**) diameters of the epithelial defect were measured using an internal ruler. The reepithelialization edge is indicated in the magnified vertical scan (**lowest image**), corresponding to the arrowed position in the middle image.

The patients were evaluated at 1, 3, and 10 days and 1 month post-surgery. In cases where the corneal epithelium had not fully healed within 3 days after surgery, an additional evaluation was performed on day 4. All follow-up examinations were conducted by the same ophthalmologist (L.W.). Postoperative examinations on days 1 and 3 prior to the removal of the BCLs included subjective questionnaires to assess ocular pain intensity and the frequency of irritation symptoms. Slit lamp examinations were performed to evaluate corneal epithelial healing, limbal and conjunctival hyperemia, BCL movement, and the presence of BCL deposits. Ocular pain intensity was measured using a numerical pain scale (NRS-11) ranging from 0 to 10 [37]. The evaluation of irritation symptoms included questions about photophobia, foreign body sensation, pain, and dry eye sensation, with each symptom graded from 0 to 4 (0 indicating never, 1 indicating sometimes, 2 indicating half of the time, 3 indicating most of the time, and 4 indicating all of the time). Objective evaluation of corneal epithelial healing was conducted using optical coherence tomography (OCT) in corneal linear scan mode. The area of the largest defect was calculated using the formula s = π(a + b)/42, where “a” represented the horizontal diameter and “b” represented the vertical diameter (Figure 1) [38]. Conjunctival and limbal hyperemia were graded on a scale from 0 to 4, with 0 indicating normal and 4 indicating severe hyperemia. Lens movement and deposits were also graded on a scale from 0 to 4 (Table 2 and Table 3) [39,40]. Once the corneal epithelium had fully healed, the healing time was recorded, and the BCLs were removed. Uncorrected (UDVA) and corrected (CDVA) distance visual acuity were assessed after BCL removal on day 3 or 4, as well as on day 10 and at 1 month postoperatively.

Protein deposition on the lenses was measured as follows: Each bandage contact lens (BCL) was collected after removal and placed in a 2 mL polypropylene centrifuge tube. A 1.5 mL extraction solution, comprising a 1:1 mixture of 0.2% trifluoroacetic acid and acetonitrile, was added. Tubes were rotated on a long-axis rotary mixer for 24 h at room temperature. Afterward, the BCL was removed, and the protein extracts were freeze-dried. To redissolve the protein, a 30-μL 0.01-M phosphate-buffered saline (PBS) with a pH of 7.4 was used. Total protein concentration on the lenses was determined using a Pierce™ BCA Protein Assay Kit (Thermo Scientific, Waltham, MA, USA).

SPSS 21.0 software (SPSS Inc., Chicago, IL, USA) was utilized for data analysis. The statistical analysis in this study was conducted using means and standard deviations (SDs) and paired-sample *t*-tests. Frequency distributions and Wilcoxon signed-rank sum tests were employed for statistical analysis involving graded counting data. Spearman correlation analysis was also utilized. A significance level of *p* < 0.05 was considered statistically significant.

## 3. Results

Table 4 presents the gender distribution, mean age, mean preoperative spherical equivalent refraction (SER), preoperative mean uncorrected distance visual acuity (UDVA) and corrected distance visual acuity (CDVA) in logMAR, optical and total laser ablation zone diameters, as well as the ablation depth.

### 3.1. Ocular Pain Intensity

**Figure 2:**
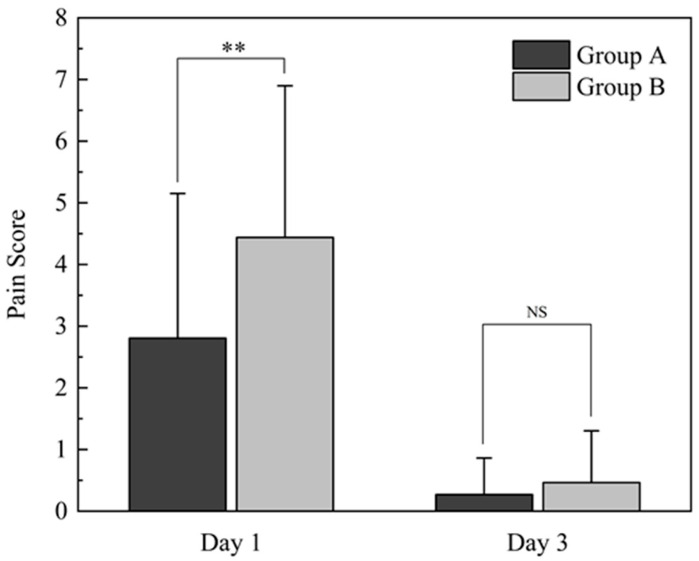
The mean pain intensity of groups A and B on days one and three postoperatively. ** *p* < 0.01 when comparing Group A with Group B; NS: No statistically significant differences.

On the first postoperative day, patients in groups A and B reported mean pain intensity scores (NRS-11) of 2.80 ± 2.35 and 4.44 ± 2.46, respectively (*p* = 0.003). By the third postoperative day, the average pain intensity had decreased to 0.27 ± 0.59 and 0.46 ± 0.84 (*p* = 0.110), respectively (Figure 2).

### 3.2. Subjective Ocular Irritation

One day after surgery, 70.7% of eyes in Group A reported a foreign body sensation intensity grade of 2 or less, compared to 31.7% of eyes in Group B (*p* = 0.001). Similarly, 53.7% of eyes in Group A reported a pain frequency grade of 2 or less, while 31.7% of eyes in Group B reported the same (*p* = 0.001). However, there were no significant differences observed between groups in photophobia or dry eye between the two groups. By the third day postoperatively, 85.4% of eyes in Group A reported a foreign body sensation intensity grade of 1 or less, compared to 58.5% of eyes in Group B (*p* < 0.001). There were no significant differences in other subjective symptoms between the two groups (Table 5). Within-group comparisons showed there was a significant improvement in photophobia, foreign body sensation, and pain symptoms from day 1 to day 3 (Group A: *p* < 0.001, *p* = 0.003, *p* < 0.001, respectively; Group B: *p* < 0.001 for all).

### 3.3. Corneal Epithelial Healing

One day after surgery, the mean corneal epithelial defect area was 14.27 ± 4.09 mm^2^ in Group A and 15.09 ± 4.95 mm^2^ in Group B (*p* = 0.20). The average epithelial healing time in Group A compared to Group B was 3.10 ± 0.30 vs. 3.12 ± 0.33 days (*p* = 0.71).

### 3.4. Slit-Lamp Evaluation

There were no statistically significant differences in limbal or conjunctival hyperemia, lens mobility, or lens deposit sores between groups A and B on days 1 to 3 postoperatively (Table 6). The mobility of lenses in both groups A and B increased from days 1 to 3 after surgery (*p* < 0.001 for Group A, *p* = 0.001 for Group B). Additionally, there was a significant reduction in limbal and conjunctival hyperemia in both groups A and B from days 1 to 3 (*p* < 0.001 for both). However, there were no significant changes in the lens deposit levels for groups A and B (*p* = 0.09 and *p* = 0.32, respectively).

### 3.5. Visual Acuity

UDVA was assessed after BCL removal on day 3 or 4 after surgery, as well as on day 10 and at 1 month postoperatively; the UDVA values (logMAR) in Group A were 0.39 ± 0.15, 0.13 ± 0.10, and 0.07 ± 0.08, respectively. In Group B, the corresponding UDVA values were 0.35 ± 0.15, 0.11 ± 0.09, and 0.06 ± 0.09, respectively. Similarly, the CDVA values in Group A were 0.28 ± 0.15, 0.07 ± 0.07, and 0.03 ± 0.06 at the same time points, while in Group B, the CDVA values were 0.27 ± 0.15, 0.07 ± 0.07, and 0.02 ± 0.05. There were no statistically significant differences in UDVA or CDVA.

### 3.6. Protein Precipitation Measured on BCL

**Figure 3:**
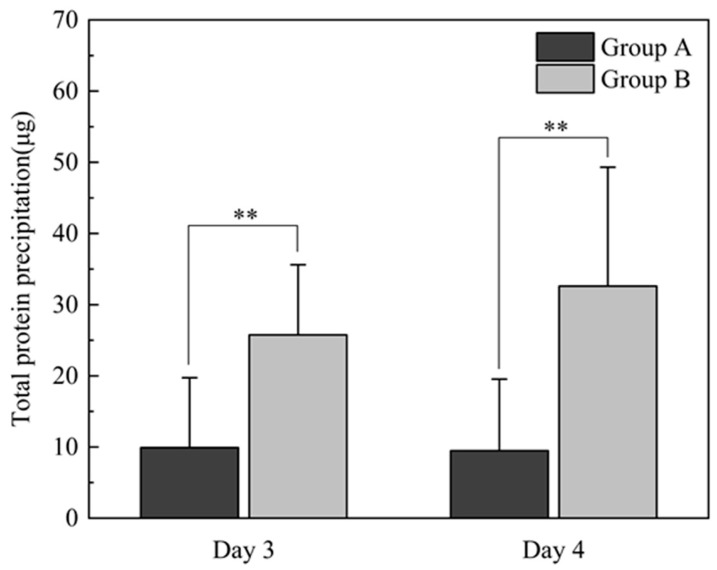
Total protein precipitation in groups A and B. ** *p* < 0.01 when comparing Group A with Group B.

A total of 66 BCLs were removed 3 days after surgery, while 16 BCLs were removed 4 days after surgery. The total protein depositions for groups A and B were 9.92 ± 9.82 μg and 25.75 ± 9.86 μg, respectively, in the lenses removed 3 days after surgery (*p* < 0.001). For the lenses removed 4 days after surgery, the total protein deposition measures were 9.47 ± 10.06 μg for Group A and 32.60 ± 16.71 μg for Group B (*p* = 0.005) (Figure 3). There was no significant difference in total protein deposition within Group A and Group B when comparing day 3 versus day 4 removal (*p* = 0.91, *p* = 0.14).

### 3.7. Correlation Analysis

Minimal correlation was observed between ocular pain intensity and lens movement (Group A: r = 0.17; Group B: r = 0.03), corneal epithelial defect area (Group A: r = −0.19; Group B: r = −0.10), or limbal and conjunctival congestion (Group A: r = −0.10 and r = −0.17, respectively; Group B: r = −0.27 and r = −0.20, respectively). Additionally, no significant correlation was found between total protein deposition and foreign body sensation (Group A: r = 0.37; Group B: r = 0.03) or lens movement (Group A: r = −0.24; Group B: r = 0.17).

## 4. Discussion

In this study, eyes fitted with Lotrafilcon A (AirOptix Night & Day) BCLs showed superior outcomes in terms of ocular pain reduction, decreased foreign body sensation, and lower protein deposition compared to eyes fitted with Balafilcon A (PureVision) BCLs following TPRK.

We found the ocular pain reported in Group A (AirOptix Night & Day) was significantly lower than in Group B (PureVision) (*p* = 0.005). This difference may be attributed to the higher Dk/t (oxygen transmissibility relative to lens thickness) value of 175 in AirOptix Night & Day compared to 101 in PureVision, as well as the smoother edge design of the former. Earlier studies have demonstrated that bandage contact lenses from different brands, utilizing various silicone hydrogel materials, provide varying levels of pain control when used following different types of surface ablation procedures. Mohammadpour et al. [41] conducted a comparative study on Lotrafilcon B and Balafilcon A silicone hydrogel bandage contact lenses for reducing pain and discomfort after photorefractive keratectomy (PRK). Their study concluded that Lotrafilcon B provided more significant relief of pain and foreign body sensation within the first 24 h postoperatively, which may be attributed to its higher oxygen permeability (Dk/t = 110) and lower material hardness. Qu et al. [42] also proposed that the design of the lens-edge contour of the bandage contact lens may influence pain control during corneal healing, in addition to the oxygen transmissibility of the BCL. Blackmore [43] suggested that an ideal lens edge should be smooth and gradual to reduce friction on the exposed nerve endings and regenerating epithelial cells, thereby providing better pain relief. Previous studies have indicated that Acuvue Oasys has the thinnest and sharpest edges, PureVision has the thickest and bluntest edges, and AirOptix Night & Day has a thick edge with a sharp fusion point between the front and back surfaces [28]. Grentzelos et al. [44] proposed that larger epithelial defects may be associated with greater pain due to the exposure of nerve endings. In our study, we did not observe significant variations in corneal epithelial defect areas between the eyes, likely due to the relatively small reepithelialization diameter in TPRK. Additionally, no significant differences in the corneal epithelial defect area or corneal healing time were observed between groups A and B on the first postoperative day, which is consistent with the findings of Mukherjee et al. [30]. It has been demonstrated that a high oxygen transmissibility (Dk/t) can effectively decrease the incidence of corneal hypoxia and edema during the healing process, as well as expedite epithelial regeneration [18,45]. In addition, stable lens fit and proper centration also contribute to the process of epithelial healing [46]. This suggests that despite the difference in oxygen transmissibility between the two lenses, both lenses are characterized by high oxygen permeability and can adequately meet the physiological requirements for corneal healing following surgery.

Our study showed that Lotrafilcon A (AirOptix Night & Day) BCLs provided superior postoperative ocular comfort to Balafilcon A (PureVision) BCLs, yet the two groups had no statistically significant difference in postoperative visual rehabilitation. Razmjoo et al. [47] reported that Senofilcon A (Acuvue Oasys) provided better control of ocular pain and discomfort after PRK compared to Lotrafilcon A (AirOptix Night & Day). Taylor et al. [28] conducted a study comparing three types of silicone hydrogel BCLs after PRK and found that Acuvue Oasys was associated with the lowest pain levels, followed by AirOptix and PureVision. Both studies indicate that while the superior comfort of Senofilcon A may be attributed to its higher water content (38%), which could reduce mechanical irritation and enhance corneal surface compatibility, no statistically significant differences were observed among these three bandage contact lenses in early visual outcomes or reepithelialization rates. This finding is congruent with the results of our study, wherein the superior postoperative ocular comfort provided by one category of bandage contact lens exhibited no correlation with divergent visual outcomes. Collectively, these data suggest that enhanced ocular comfort is not statistically associated with postoperative visual acuity recovery and does not exert a significant facilitatory effect on epithelial healing. Yakar et al. [48] also compared the efficacy of Lotrafilcon A and Senofilcon A bandage contact lenses in terms of visual rehabilitation and ocular discomfort following photorefractive keratectomy (PRK) and had different results. They found eyes fitted with senofilcon A BCLs demonstrated better postoperative visual rehabilitation, while ocular discomfort scores did not differ significantly after PRK. Their study diverged from the findings suggested by Razmjoo et al. and Taylor et al. in terms of ocular discomfort. This discrepancy may be attributed to the subjective nature of ocular discomfort or to inter-racial differences. In terms of visual rehabilitation, Razmjoo et al. compared uncorrected visual acuity on postoperative day 3 and found no difference between the two BCLs. While visual rehabilitation was objectively measured at postoperative 15 days and 1 month by Yakar et al. Their finding that senofilcon A led to better results in visual rehabilitation than lotrafilcon A may be due to the fact that we measured refractive status with an objective method and evaluated it at a later time point.

Lim et al. [46] suggested that BCLs should demonstrate adequate mobility to prevent lens adherence, enhance patient comfort, improve stability, and promote proper centration, thereby facilitating corneal wound healing. Some studies have indicated that BCLs with high water content may have a greater tendency to adhere to the corneal epithelium [40], and that such BCLs may gradually tighten progressively with extended wear [42]. In our study, the movement of the BCLs on the first day after surgery ranged between levels 0 and 2 for all eyes except one, and there was no significant difference in lens movement between the two groups, which is consistent with the findings of Fonn et al. [49]. However, on day 3 postoperatively, there was a slight increase in lens movement in both groups compared to the first day, likely due to resolution of early corneal edema and decreased ocular irritation, which may have affected blinking and, in turn, influenced lens movement.

Deposits on contact lenses typically include protein, lipid, microorganisms, and calcium salts, which accumulate on the lens surface and within its matrix. Among these precipitates, protein deposition is especially significant [50], as it can lead to ocular discomfort, reduced visual acuity, and increased inflammation [51]. In our study, the AirOptix Night & Day BCL belongs to type I lenses (the U.S. FDA classifies soft contact lenses into four categories based on their water content and ionization degree: non-ionic types I and II, with water content <50% and >50%, respectively, and ionic types III and IV, with water content <50% and >50%, respectively), which have a plasma coating forming a permanent ultra-thin (25 nm) hydrophilic surface and a water content of 24%. In contrast, the PureVision BCL belongs to type III silicon hydrogel lenses with a negative charge and a water content of 36%. The significantly lower protein deposition in Group A compared with Group B indicates that the AirOptix Night & Day lens exhibits greater resistance to protein buildup than PureVision lens, which is consistent with the observation of Sack et al. [52] These authors measured protein precipitation on various soft contact lenses and found that the thickness of surface precipitation on type IV lenses was considerably greater than that on type I lenses. Myers et al. [53] analyzed type I and IV lenses and discovered that the total surface deposition was similar between the two types, but the total protein precipitation on type IV lenses was nearly 40 times higher than that on type I lenses. Therefore, it appears that protein precipitation on the surface and inside of soft contact lenses is positively correlated with water content and is more pronounced in ionic contact lenses.

While correlation analysis did not reveal significant correlations between lens protein deposition and subjective ocular discomfort (pain: Group A r = 0.37, Group B r = 0.03; foreign body sensation: Group A r = 0.37, Group B r = 0.03), the distinct advantages of Lotrafilcon A—reduced pain, minimized foreign body sensation, and diminished protein deposition—collectively align with patient-centered care and synergistically improve TPRK postoperative outcomes. Consistent with our results, Lotrafilcon A markedly reduced acute postoperative pain on day 1 (2.80 ± 2.35 vs. 4.44 ± 2.46, *p* = 0.003) and alleviated foreign body sensation: 70.7% of Group A eyes had a foreign body sensation grade ≤2 on day 1 (vs. 31.7% of Group B, *p* = 0.001), and 85.4% achieved grade ≤1 by day 3 (vs. 58.5% of Group B, *p* < 0.001). These improvements relieve postoperative distress, preserve daily function (e.g., visual engagement, rest quality) and enhance treatment satisfaction—key tenets of patient-centered refractive care. Simultaneously, Lotrafilcon A showed substantially lower protein deposition (day 3: 9.92 ± 9.82 μg vs. 25.75 ± 9.86 μg, *p* < 0.001; day 4: 9.47 ± 10.06 μg vs. 32.60 ± 16.71 μg, *p* = 0.005), clinically meaningful as protein accumulation links to prolonged irritation, visual blurring, and elevated inflammation risk. Despite no direct correlations between these outcomes, their combined effects create a more favorable postoperative environment: reduced pain/foreign body sensation relieves immediate discomfort, while less protein deposition lowers delayed adverse risks, collectively enhancing recovery-phase quality of life. This comprehensive benefit aligns with core patient-centered principles, prioritizing both objective therapeutic goals and subjective comfort, functional integrity, and overall treatment experience.

The main limitations of this study are the small sample size and the strong subjective factors of the observation indicators. Although all postoperative evaluations were performed by a single examiner to reduce variability, the subjective nature of symptom grading—combined with the severity of ocular irritation in the early postoperative period—may have introduced bias, particularly in evaluating hyperemia, lens mobility, and lens deposits. Additionally, the follow-up timing was not strictly standardized, which may have affected the consistency of measurements such as epithelial defect size and pain intensity on postoperative day 1. Future studies should aim to include a larger sample size, adopt standardized follow-up intervals, and incorporate quantitative imaging techniques for congestion analysis. Laboratory assays for lens-associated lysozyme and lipid deposition may also help validate surface biofouling findings with greater precision.

## 5. Conclusions

Based on the findings of this study, Air Optix Night & Day (Lotrafilcon A) bandage contact lenses demonstrated superior performance compared to PureVision (Balafilcon A) lenses when used after TPRK. Specifically, Lotrafilcon A lenses were more effective in reducing ocular pain and foreign body sensation and showed significantly lower levels of protein deposition. These results suggest that Lotrafilcon A may be a more suitable option for therapeutic use following TPRK.

## Figures and Tables

**Table 1:** Characteristics of two silicone hydrogel lenses used in this study.

Parameter	BCL A (Air Optix Night & Day)	BCL B (PureVision)
Material	Lotrafilcon A	Balafilcon A
Water content	24%	36%
Dk	140	91
Dk/t	175	101
Surface treatment	Plasma Coating	Plasma Oxidation
Elastic modulus	1.4 MPa	1.1 MPa
Central thickness	0.08 mm	0.09 mm
BOZR	8.6 mm	8.6 mm
TD	13.8 mm	14 mm
Continuous overnight wear	28 days	21 days

Dk: Oxygen permeability (×$10^{-11}$); Dk/t: Oxygen transmissibility (×$10^{-9}$); BOZR: Back optic zone radius; TD: Total diameter; BCL: Bandage contact lens.

**Table 2:** Lens movement grading scale.

Grade	Lens Movement
0	Extremely inadequate; lens does not move on blinking
1	Slightly inadequate; lens moves < 0.2 mm on blinking
2	Optimum; lens moves between 0.2 and 0.4 mm on blinking
3	Slightly excessive; lens moves between 0.4 and 1.0 mm on blinking
4	Extremely excessive; lens moves > 1.0 mm on blinking

**Table 3:** Lens surface deposits grading scale.

Grade	Lens Deposits
0	None
1	Five or fewer small (<0.1 mm) deposits or very slight film covering up to 25% of the lens surface
2	More than five small individual deposits, one individual deposit 0.1 to 0.5 mm in diameter, or film covering between 25 and 50% of the lens surface area
3	Multiple deposits between 0.1 and 0.5 mm in diameter, one deposit larger than 0.5 mm in diameter, or moderate film covering between 50 and 75% of the lens surface area
4	Multiple deposits of 0.5 mm in diameter or larger or film covering more than 75% of the lens surface area

**Table 4:** Patients’ preoperative and surgical parameters.

	Group A	Group B	*p*
Gender (n)	Male/female = 18/23		
Age (y)	25.46 ± 4.36 (range: 18–35)		
SER (D)	−5.53 ± 1.94	−5.51 ± 2.00	0.747
Mean UDVA (logMAR)	1.08 ± 0.30	1.10 ± 0.34	0.315
Mean CDVA (logMAR)	−0.00 ± 0.01	−0.01 ± 0.02	0.157
Diameter of the operative Optical zone (mm)	6.39 ± 0.52	6.38 ± 0.53	0.486
Diameter of the surgical Treatment zone (mm)	7.95 ± 0.37	7.94 ± 0.35	0.729
Surgical ablation depth (μm)	138.66 ± 18.10	138.27 ± 19.60	0.698

SER: Spherical equivalent refraction; UDVA: Uncorrected Distance Visual Acuity; CDVA: Corrected Distance Visual Acuity.

**Table 5:** Comparison of the frequency of subjective eye irritation symptoms between groups A and B (n, %).

	Day 1			Day 3		
Grade	Group A	Group B	*p*	Group A	Group B	*p*
Foreign body sensation			0.001 *			0.000 *
0	12 (29.3%)	4 (9.8%)		16 (39.0%)	6 (14.6%)	
1	9 (22.0%)	3 (7.3%)		19 (46.3%)	18 (43.9%)	
2	8 (19.5%)	6 (14.6%)		2 (4.9%)	6 (14.6%)	
3	6 (14.6%)	8 (19.5%)		4 (9.8%)	9 (22.0%)	
4	6 (14.6%)	20 (48.8%)		0 (0%)	2 (4.9%)	
Pain			0.001 *			0.705
0	4 (9.8%)	0 (0%)		24 (58.8%)	23 (56.1%)	
1	12 (29.3%)	9 (22.0%)		14 (34.1%)	14 (34.1%)	
2	6 (14.6%)	4 (9.8%)		1 (2.4%)	3 (7.3%)	
3	9 (22.0%)	9 (22.0%)		2 (4.9%)	1 (2.4%)	
4	10 (24.4%)	19 (46.3%)		0 (0%)	0 (0%)	
Photophobia			0.190			1.000
0	4 (9.8%)	4 (9.8%)		4 (9.8%)	4 (9.8%)	
1	3 (7.3%)	1 (2.4%)		22 (53.7%)	23 (56.1%)	
2	7 (17.1%)	7 (17.1%)		9 (22.0%)	7 (17.1%)	
3	12 (29.3%)	13 (21.7%)		5 (12.2%)	6 (14.6%)	
4	15 (36.6%)	16 (39.0%)		1 (2.4%)	1 (2.4%)	
Dry eye			0.066			0.157
0	21 (51.2%)	20 (48.8%)		16 (39.0%)	15 (36.6%)	
1	12 (29.3%)	10 (24.4%)		18 (43.9%)	18 (43.9%)	
2	4 (9.8%)	5 (12.2%)		1 (2.4%)	2 (4.9%)	
3	2 (4.9%)	3 (7.3%)		5 (12.2%)	5 (12.2%)	
4	2 (4.9%)	3 (7.3%)		1 (2.4%)	1 (2.4%)	

* *p* < 0.05 when comparing Group A with Group B.

**Table 6:** Comparison of subjective evaluation indexes under slit lamp between groups A and B (n, %).

	Day 1			Day 3		
Grade	Group A	Group B	*p*	Group A	Group B	*p*
Limbal hyperemia			0.157			0.257
0	0 (0%)	0 (0%)		2 (4.9%)	1 (2.4%)	
1	11 (26.8%)	8 (19.5%)		31 (75.6%)	30 (73.2%)	
2	24 (58.5%)	26 (63.4%)		8 (19.5%)	10 (24.4%)	
3	6 (14.6%)	7 (17.1%)		0 (0%)	0 (0%)	
4	0 (0%)	0 (0%)		0 (0%)	0 (0%)	
Conjunctival hyperemia			0.414			0.705
0	0 (0%)	0 (0%)		4 (9.8%)	4 (9.8%)	
1	9 (22.0%)	7 (17.1%)		29 (70.7%)	28 (68.3%)	
2	25 (61.0%)	27 (65.9%)		8 (19.5%)	9 (22.0%)	
3	7 (17.1%)	7 (17.1%)		0 (0%)	0 (0%)	
4	0 (0%)	0 (0%)		0 (0%)	0 (0%)	
Lens movement			0.527			0.132
0	30 (73.2%)	31 (75.6%)		11 (26.8%)	16 (39.0%)	
1	7 (17.1%)	6 (14.6%)		21 (51.2%)	16 (39.0%)	
2	3 (7.3%)	4 (9.8%)		9 (22.0%)	9 (22.0%)	
3	1 (2.4%)	0 (0%)		0 (0%)	0 (0%)	
4	0 (0%)	0 (0%)		0 (0%)	0 (0%)	
Lens deposits			0.796			0.071
0	8 (19.5%)	4 (9.8%)		8 (19.5%)	3 (7.3%)	
1	15 (36.6%)	21 (51.2%)		25 (61.0%)	28 (68.3%)	
2	17 (41.5%)	16 (39%)		8 (19.5%)	10 (24.4%)	
3	1 (2.4%)	0 (0%)		0 (0%)	0 (0%)	
4	0 (0%)	0 (0%)		0 (0%)	0 (0%)	

## Data Availability

The study was registered in the Chinese Clinical Trial Registry on 7 December 2017, and the registration number is ChiCTR-IOR-17013751.

## Abbreviations

The following abbreviations are used in this manuscript:

BCLs	Bandage contact lenses
TPRK	Transepithelial photorefractive keratectomy
UDVA	Uncorrected Distance Visual Acuity
CDVA	Corrected Distance Visual Acuity
PRK	Photorefractive keratectomy
LASIK	Laser in situ keratomileusis
FDA	Food and Drug Administration
SER	Spherical equivalent refraction
